# Visual Search Test for Residents Chronically Exposed to Methylmercury in the Minamata Area

**DOI:** 10.3390/toxics13080657

**Published:** 2025-07-31

**Authors:** Shigeru Takaoka, Kenta Matsunaga

**Affiliations:** Kyoritsu Neurology and Rehabilitation Clinic, 2-2-28 Sakurai-cho, Minamata 867-0045, Japan

**Keywords:** Minamata disease, cognitive function, visual search, reaction time, methylmercury

## Abstract

In individuals exposed to relatively mild methylmercury, even if they appeared to be independent in activities of daily living (ADL), slower judgment and motor responses in daily activities were observed, suggesting potential cognitive impairment. To quantitatively assess this impairment, we measured reaction time (RT) in a visual search test, as a visual cognitive ability test. The study participants included 24 residents from contaminated areas with sensory impairments in the limbs but no visual field defects (E group), as well as 12 individuals from non-contaminated areas (Group C). The 24 participants from contaminated areas were further divided into two groups: 12 without hand motor coordination disorders (Group E-HA) and 12 with such disorders (Group E+HA). Participants were instructed to search for the target letter “Z” on a computer screen, and the visual stimuli consisted of two, six, or ten alphabet letters. An equal number of trials contained “Z” and did not contain “Z,” for a total of thirty trials, which were conducted twice. RT was significantly longer in Group E+HA, followed by Group E-HA, and then Group C. However, in the second test, RT decreased in all cases, with a greater reduction in the exposed groups compared to the control group. These results suggest that methylmercury exposure may cause cognitive impairment, yet it also possesses plasticity.

## 1. Introduction

Minamata disease is caused by ingesting fish and/or shellfish contaminated with methylmercury. It was first discovered in Japan in 1956. The Chisso Company used mercury to produce acetaldehyde and drained the toxic wastewater from the process into the sea for 36 years (1932–1968). Soon after its discovery, the neurological abnormalities of local inhabitants, including Hunter–Russell syndrome, attracted attention [1]. These neurological symptoms are known to affect patients throughout the Yatsushiro Sea coast [2].

However, methylmercury affects not only the neurological system but also psychological systems, including intelligence, personality, and psychiatric findings. In Minamata, Inoue et al. reported on the psychiatric abnormalities observed in patients with severe illness [3]. Most of these patients also had distinct abnormalities in motor and sensory functions. Since then, patients with milder cases of Minamata disease have been found in Minamata exhibiting only somatosensory disturbances, such as neurological abnormalities [4]. Many patients with mild neurological symptoms appear to have impaired judgment and motor skills in daily life, which suggests cognitive decline. However, there are very few studies on psychiatric or psychological abnormalities in milder cases of Minamata disease.

Since the 1990s, the effects of exposure to lower concentrations of methylmercury in children have attracted attention. Psychomotor performance in children born to mothers exposed to lower concentrations of methylmercury (approximately 10 µg/g [=ppm] in hair) has been measured [5]. We investigated the visual search function, one of the cognitive functions, in patients aged 30 to 50 who had the possibility of having been exposed to methylmercury during fetal development and after birth. These patients exhibited milder symptoms of Minamata disease, as reported by Inoue.

## 2. Materials and Methods

### 2.1. Subjects

The study subjects (Group E) consist of residents of contaminated areas who were born and raised in the Yatsushiro Sea coastal region. They regularly consumed seafood from the Yatsushiro Sea between 1950s and 1970s. They underwent testing for Minamata disease at Minamata Kyoritsu Hospital or an adjacent clinic and were diagnosed with sensory disturbances in the limbs but not with visual field constriction.

Methylmercury exposure in Minamata was widespread and severe, yet only a limited number of mercury measurements were taken. The extent of exposure was estimated based on seafood consumption rates and symptoms observed in people with high seafood intake. Many studies have used this method [2,4,6,7,8].

The survey was conducted from November 2007 to January 2008 and from August to September 2008. Twenty-six participants were involved in this study, with an average age of 49.7 ± 7.6 years. They may have been exposed to methylmercury both before and after birth, but the proportion was unclear.

The control subjects (Group C) were all non-fishermen from other industries who were born in the vicinity of Kumamoto City and had no history of living in the Minamata area. In January, April, and September 2008, 13 subjects (aged 47.7 ± 7.4 years) with no history of living in contaminated areas were surveyed.

Because of the difference in age between Groups C and E, the subjects in each group were rescreened according to the criteria outlined in Section 2.2.

Additionally, we examined their educational background and daily computer usage. We compared the occupations of the subjects and their parents within each group.

### 2.2. Standard Neurological Examination

In Group E, standard neurological examinations and two-point discrimination tests were performed. These examinations investigated visual field abnormalities, hearing impairment, speech disorders, and motor coordination disorders of the upper and lower limbs and trunk. They also investigated superficial sensory disorders, such as touch and pain perception. The two-point discrimination test used the tongue (or lower lip) and the tips of both index fingers.

These tests were conducted using the same methods as in previous studies [7]. Superficial sensory disorders were examined using pins and brushes. Two-point discrimination was tested using a draft divider. The examining physician evaluated other tests, including visual field and hearing impairments, without the use of special equipment. Visual field narrowing was diagnosed when lateral visual acuity was 80° or less during a straight-ahead visual field test. Trunk ataxia was assessed using normal walking, tandem walking, and single-leg balance tests. Lower limb dyskinesia was diagnosed using the heel–shin test. Based on these results, the physician classified subjects as having severe, moderate, mild, or normal conditions.

The 26 individuals who were exposed were classified based on the presence or absence of finger motor dysfunction. Those with mild bilateral impairment or obvious unilateral impairment in the “touch nose” test or dyadochokinesis were classified as the E+HA group. Those with no or minimal motor dysfunction were classified as the E-HA group. As a result, Group E was reclassified into Groups E-HA (13 participants, average age 49.5 ± 6.3 years) and E+HA (13 participants, average age 49.8 ± 9.0 years).

After reclassifying Group E into Group E-HA and Group E+HA, we excluded the youngest individual from Group C and the oldest individual from each of Group E-HA and Group E+HA to minimize age differences among the three groups. The final analysis included three groups: Group C (12 participants, age 48.6 ± 7.0 years, 33–58 years), Group E-HA (12 participants, age 48.8 ± 6.0 years, 40–56 years), and Group E+HA (12 participants, age 48.8 ± 8.5 years, 34–58 years). No neurological examinations were performed on the control subjects (Group C), and none of the study participants reported neurological disorders or difficulties in daily living.

### 2.3. Reaction Time (RT) Measurement

One method of measuring visual cognition ability is visual search. Visual search is the measurement of a person’s capacity to find a target hidden among distractors. In order to measure visual search capacity, a personal computer was used. A stimulus is shown on the computer display, and the reaction input is carried out by using the keyboard or other methods.

The computers used to measure the RT were PC laptops running Windows XP or Windows Vista and were equipped with touchpads, which we used for the input reaction. The installed visual search application was a commercially available product from Nakanishiya Publishing in Kyoto, Japan. It was featured in the Japanese book “Cognitive Psychology Experiments Using a Personal Computer” [9].

**Figure 1:**
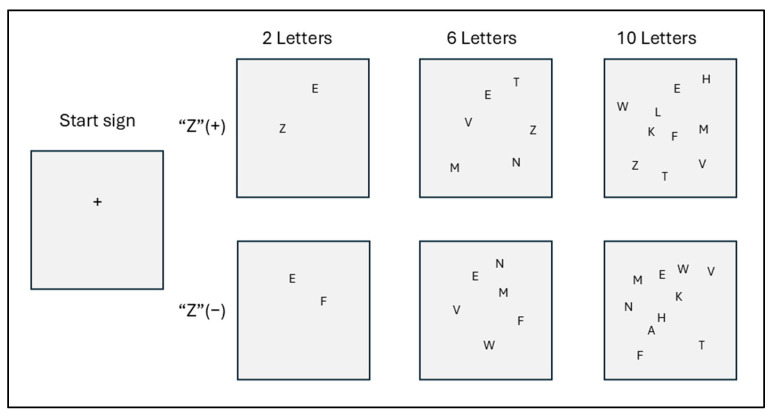
Stimulus screen (samples).

Figure 1 shows a sample of the stimulus screen. The target is the letter “Z,” and the distractors were selected from letters written with straight lines rather than curves. For instance, rather than D, O, or B, the distractors were A, V, K, or M.

The visual stimuli on the computer screen displayed two, six, or ten letters. There were two variations of each: half included the target “Z,” and half did not. Each screen was displayed randomly ten times, resulting in a total of thirty visual stimulus tests. The purpose of these tests was to find the letter “Z” as quickly as possible on each screen.

Two touchpads were used, and subjects were instructed to press the left touchpad when a “Z” appeared and the right touchpad when a “Z” did not appear. A plus sign (+) appeared on the screen for 500 milliseconds to indicate the start of the test. This was followed by a blank screen for 1000 milliseconds. Then, a stimulus appeared on the screen, and the subjects were instructed to press the appropriate touchpad as quickly as possible. The stimulus screen remained on until the subject touched the touchpad. It was not turned off during the test. The stimuli were presented randomly, and the RTs were measured six times for each part of the test.

The complete test was conducted twice. The second test was conducted 10 min after the first. If this was not possible, the second test was conducted one or two days after the first test.

### 2.4. Statistical Methods

All calculations were performed using Stata, version 14 (Lightstone Corp., Tokyo, Japan). We performed a chi-square analysis to compare the subjects’ educational backgrounds, daily computer usage times, and two-point discrimination results.

When comparing the average RTs, we tested the normality of 36 data groups (3 groups × 3 types of stimuli × 2 (presence or absence of Z) × 2 (number of trials)) using the Shapiro–Wilk test. Normality was found in 32 groups except for 4 groups, so we performed a *t*-test rather than a nonparametric test to compare the mean values. Paired *t*-tests (two-tailed) were used to compare mean RTs within groups, and unpaired *t*-tests (two-tailed) were used to compare mean RTs between different groups.

## 3. Results

### 3.1. Basic Information

Basic information is shown in Table 1. The three groups were roughly the same age. Similarly, they spent the same amount of time using computers each day. Group C had a slightly higher educational level, but the difference was not significant. Of the six individuals in Group E+HA and eleven individuals in Group E-HA for whom both the individuals’ and their parents’ occupations could be identified, one individual in Group E-HA, one individual in Group E+HA, and no individuals in Group C were engaged in fishing-related work.

### 3.2. Neurological Findings

All subjects in the exposed groups had four-limb or generalized somatosensory disturbance. Other neurological symptoms are listed in Table 2. There were no symptoms of visual constriction or hearing loss. Dysarthria was present in only one subject from Groups E-HA and E+HA, respectively. Most upper extremity ataxia was mild. Severe truncal ataxia was observed in four subjects in Group E-HA and nine subjects in Group E+HA.

Table 3 shows the number of subjects with a two-point discrimination threshold of 5 mm or more. Two-point discrimination was worse in Group E+HA than in Group E-HA. The findings in Table 2 and Table 3 indicate that, overall, the E+HA group had more severe motor and sensory impairments than the E-HA group.

### 3.3. Reaction Time (RT)

**Figure 2:**
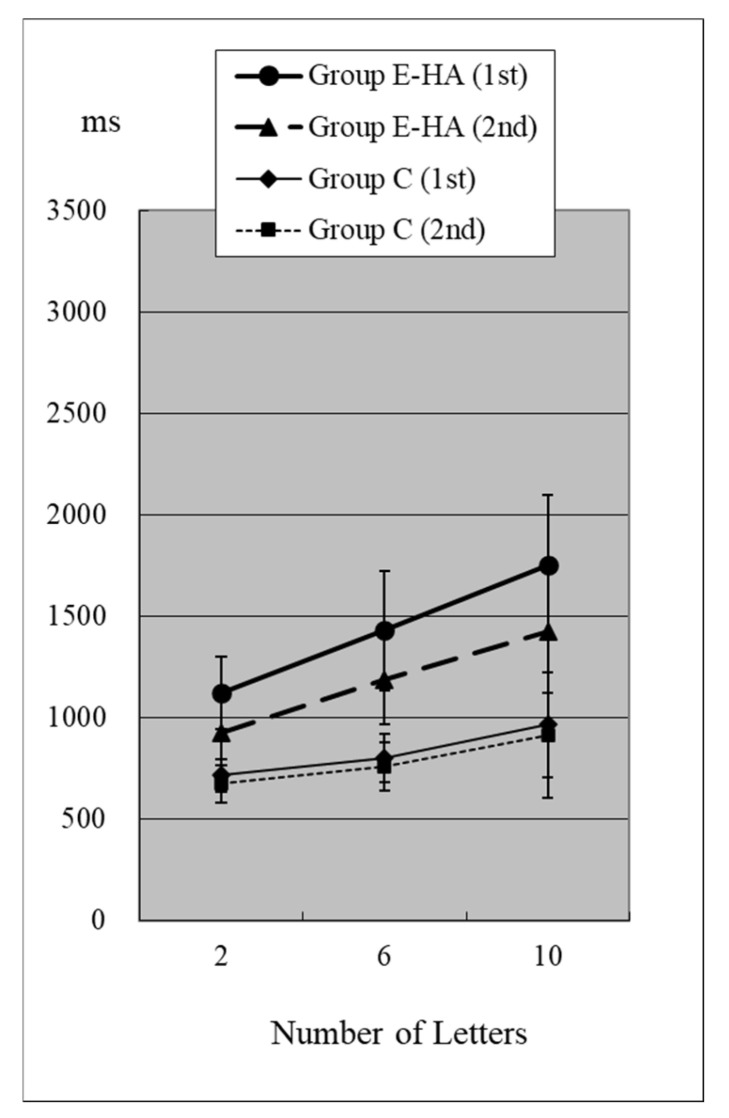
RT of Group C/E-HA (“Z”+).

**Figure 3:**
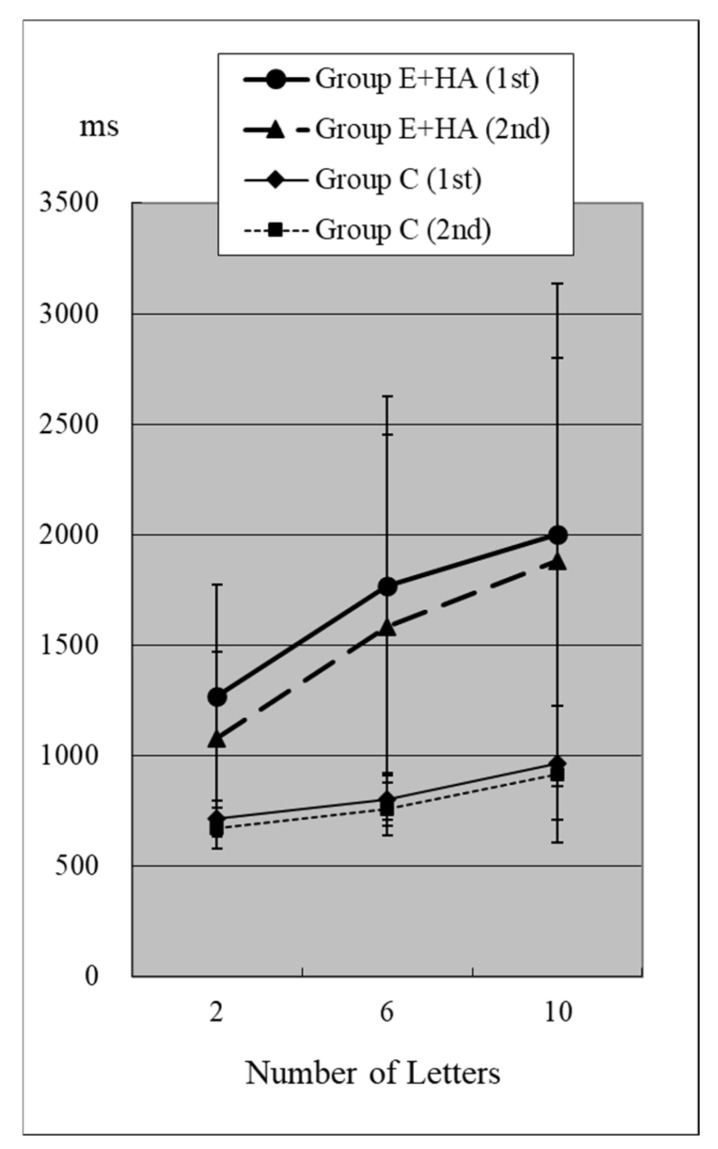
RT of Group C/E+HA (“Z“+).

**Figure 4:**
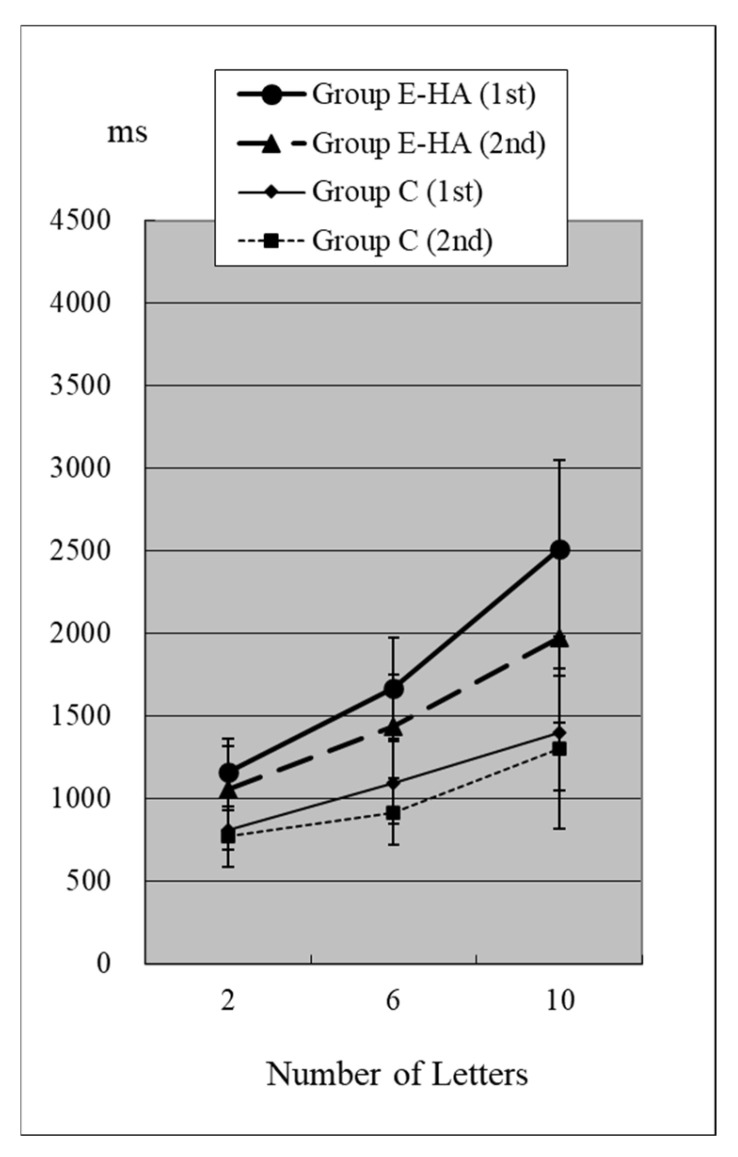
RT of Group C/E-HA (“Z“−).

**Figure 5:**
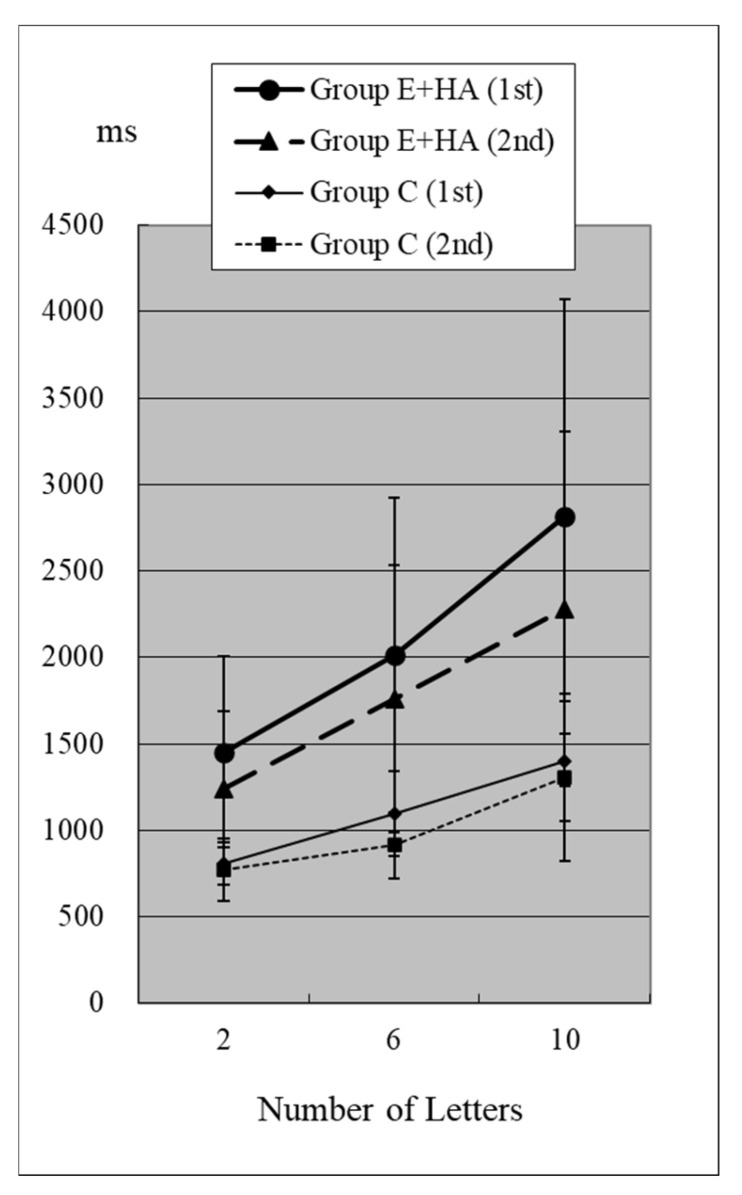
RT of Group C/E+HA (“Z“−).

Reaction times (RTs) when the target was present are shown in Figure 2 and Figure 3 and Table S1. RTs when the target was absent are shown in Figure 4 and Figure 5 and Table S2. Table 4 shows the number of errors in the responses to each test. On average, each group had around one error, and there was no significant difference between groups.

The *p*-value comparisons for each test can be found in Table S3 (difference in number of letters), Table S4 (presence/absence of target “Z”), Table S5 (first/second test), and Table S6 (among Groups C, E-HA, and E+HA).

In Group C’s first test, RTs were 715 ± 81 ms (two letters), 803 ± 118 ms (six letters), and 967 ± 259 ms (ten letters), respectively (Figure 2, Table S1). As the number of presented letters increased, RTs increased significantly (Figure 2, Table S3). This phenomenon was observed in both the first and second tests, as well as in the test of Groups E-HA and E+HA (Figure 2 and Figure 3 and Table S3).

In the first test for Group C, RT without the target “Z” was 810 ± 118 ms (two letters), 1098 ± 248 ms (six letters), and 1400 ± 348 ms (10 letters), respectively (Figure 4, Table S2). As the number of presented letters increased, RTs increased significantly (Figure 4, Table S3). RTs were significantly greater than in the first test with “Z” (Table S4). This phenomenon was observed in both the first and second tests, as well as in the test of Groups E-HA and E+HA (Figure 4 and Figure 5, Tables S3 and S4). In both Groups E-HA and E+HA, RTs significantly increased in tests with “Z” compared to tests without “Z,” except for one test (the first test in the E-HA group) (Figure 2, Figure 3, Figure 4 and Figure 5, Table S4).

In the second test with Group C, the RT with “Z” was 673 ± 93 ms for two letters, 758 ± 119 ms for six letters, and 916 ± 309 ms for ten letters, respectively (Figure 2, Table S1). In the second test, RTs tended to be shorter than in the first test, but no statistical differences were observed except for the six-letter test without “Z” (Figure 2 and Figure 4, Table S5). In Group E-HA, RTs decreased significantly in all tests except the two-letter test without “Z” (Table S5). Similarly, RTs were significantly reduced in all tests except for the 6- and 10-letter tests with “Z” in Group E+HA (Figure 3 and Figure 5, Table S5).

When comparing the three groups, in the first test with “Z”, RTs were 715 ± 81 ms (Figure 2 and Figure 3, Group C), 1122 ± 179 ms (Group E-HA), and 1271 ± 505 ms (Group E+HA). RTs were shortest in the following order: Group C, Group E-HA, and Group E+HA (Table S6). Significant differences were observed between Groups C and E-HA, as well as between Groups C and E+HA. However, no significant differences were found between Groups E-HA and E+HA (Table S6). The same phenomenon was observed in the test without “Z,” with significant differences between Groups C and E+HA but not between Groups E-HA and E+HA (Table S6).

## 4. Discussion

Exposure to methylmercury is known to cause neurological symptoms, including damage to the cerebral and cerebellar cortices; somatosensory disorders; narrowing of the visual field; ataxia; dysarthria; hearing impairment; and tremors [1]. However, methylmercury causes not only neurological abnormalities but also psychological and psychiatric abnormalities.

In 1963, 43 patients with severe Minamata disease were reported to exhibit psychiatric abnormalities alongside clear neurological symptoms [3]. All patients exhibited ataxia and dysarthria. Peripheral sensory disorders were present in 30 cases (70%), while visual and hearing impairments were present in 29 cases each (67%). Eight cases (19%) exhibited primitive reflexes. Intellectual disability and personality disorders were observed in all cases. Harada also reported intellectual disability in 46% of family members of patients with acute or subacute Minamata disease [1].

However, as the severity of the poisoning symptoms decreases, the mental symptoms also become milder. In 1971, a survey targeting the entire population of Minamata City and its surrounding areas was conducted [10]. The survey found that, in Minamata’s high-exposure areas, 20.4% of the population exhibited intellectual disabilities, and 12.5% exhibited mood and behavioral disorders. However, these outcomes were not assessed using psychological batteries, and standardized interview forms were not used; the diagnoses were made clinically by psychiatrists.

A survey of Katsurajima residents [4], conducted between 1974 and 1979, found that 95% of the 41 individuals born before 1945, 91% of the 11 individuals born between 1946 and 1953, 29% of the seven individuals born between 1954 and 1960, and 0% of the 20 individuals born between 1961 and 1972 reported symptoms such as “forgetfulness, difficulty with calculations, and inability to organize thoughts” on the questionnaire. Physicians diagnosed 77.4% of Katsurajima residents and 3.0% of the study area residents with emotional disorders.

Since the 1990s, epidemiological studies have examined the effects of fetal exposure to methylmercury on neuropsychological development, notably in the Faroe Islands [11,12] and the Seychelles [13,14], and numerous studies have been conducted worldwide [15,16,17]. However, studies on the effects of methylmercury exposure on mental and intellectual aspects have not been conducted in detail in Minamata and Niigata, Japan, where people were exposed to higher concentrations of mercury.

Even in surveys conducted after 2000, many Minamata disease patients reported memory loss, irritability, and anxiety [2,7]. Sato has conducted research on the personalities of Minamata disease patients [18,19].

It is known that intellectual and emotional disorders exist among residents exposed to methylmercury and among patients with Minamata disease and that the severity of neurological abnormalities correlates with the presence of these disorders. However, few studies have quantitatively measured intellectual disorders in these populations.

Since the 1980s, research institutions in Japan, such as universities, have not actively conducted clinical and epidemiological research on Minamata disease [2,8]. The health status of hundreds of thousands of non-exposed individuals has primarily been observed by a limited number of general clinicians. We recognized the importance of researching mental and intellectual disabilities, but we were largely consumed by detecting neurological abnormalities through examinations by a small number of physicians. This left us with very limited opportunities to conduct and publish such research.

One of our studies on intellectual disabilities, conducted between 2007 and 2008, involved measuring RTs. In the Minamata region, the levels of methylmercury in the hair and other samples of most residents had not been measured. Therefore, we selected individuals in the region who consumed seafood and exhibited clear neurological symptoms as study participants, as they were considered to have had a certain level of exposure. Additionally, conditions such as cerebrovascular disorders, cognitive function, and musculoskeletal issues become associated with RT as individuals age. Thus, this study primarily targeted residents in their 40s and 50s.

In this visual search test, the letter “Z” is the target stimulus, while all other letters are distractors. Therefore, it is generally expected that RTs will increase with the number of letters and when the “Z” target indicator is absent [9]. This was confirmed in all three groups (Figure 2, Figure 3, Figure 4 and Figure 5). The longest RTs were observed in Group E+HA, followed by Group E-HA, and then Group C. Additionally, the standard deviations of RTs showed that Group E+HA had the longest, followed by Group E-HA and then Group C (Tables S1 and S2). These results are believed to reflect the varying degrees of sensory and cognitive impairments associated with mild central nervous system disorders caused by methylmercury. These disorders are characterized by pathological mechanisms involving the “thinning-out” loss of neurons in the cerebral and cerebellar cortex.

The different results between Groups E-HA and E+HA may be due to finger movement disorders in the subjects or the possibility that judgment ability was more impaired in Group E+HA than in Group E-HA. Group C showed a tendency toward higher educational attainment compared to the other two groups, but this difference was not significant. This factor alone was considered insufficient to explain the differences in RTs. Therefore, it was concluded that methylmercury influences cognitive function.

As previously mentioned, the effects of low concentrations of methylmercury on human neurodevelopment have been debated since the 1990s. Studies in the Faroe Islands have found a significant correlation between methylmercury poisoning and impaired motor, attention, and language functions [11,12]. A cohort study in the Seychelles also suggested an association between methylmercury contamination and impaired child development [13,14].

Among the studies targeting these children, two types of RTs are mentioned. One is “simple reaction time (RT),” and the other is “hit reaction time (RT) in a continuous performance test.”

In a test conducted on Japanese mothers and their seven-year-old children, the average simple RT to audible stimuli was 353 ± 61 ms for boys on the right side, 373 ± 67 ms on the left side, 357 ± 50 ms for girls on the right side, and 383 ± 58 ms on the left side [20]. There was a slight correlation with maternal hair mercury levels, which were also correlated with maternal hair, but no statistically significant difference was found [11]. In a study of 14-year-olds in the Faroe Islands, the simple RT to sound was 249 ± 41.5 ms, and no correlation was observed with umbilical cord blood, maternal hair, or umbilical cord tissue. The average hit RT in the continuous performance test was 759 ms (interquartile range = 705–809 ms) for seven-year-olds [12] and 497 ± 45.5 ms for fourteen-year-olds [11], indicating a faster RT at age 14. RT was correlated with umbilical cord blood mercury concentration levels in both studies.

RTs are generally longer in continuous performance tests than in simple reaction tests because subjects must make a judgment after recognizing the stimulus. However, even continuous performance tests usually use only one stimulus. These reports were used to investigate responses to sound.

Our method uses visual stimuli rather than auditory stimuli for adults. Because there are many stimuli and subjects must recognize multiple characters, the task is more complex than continuous performance tests. Our study subjects’ RTs were 715 ± 81 ms for the first response to two characters containing a “Z” in the control group, which was longer than the simple or continuous performance times mentioned above. We believe this is due to the aforementioned reasons.

Our results indicate that exposure to methylmercury affects RT. RT, as measured by this test, reflects visual sensory, cognitive, and executive functions. It is expressed as a combination of these functions: recognizing characters on a screen through vision, recognizing “Z” among multiple characters, determining whether “Z” is present or absent, and pressing the touchpad. Additionally, the validity of the test can be evaluated using six types of RT measurement methods.

Although it is referred to as the same methylmercury poisoning, the symptoms of health disorders vary depending on the age at which exposure occurs, the duration of exposure, and the age at which an examination is performed. Our study participants were individuals chronically exposed to levels sufficient to cause moderate neurological impairment over an extended period. All participants were able to perform basic activities of daily living (ADLs), such as eating, toileting, and mobility, without assistance. However, we frequently heard reports that many of these individuals had occupational disabilities or experienced symptom fluctuations.

Compared to the first test, RTs tended to be shorter in the second test. While there was no significant difference in Group C, RTs were significantly shorter in Groups E-HA and E+HA in the second test. Thus, cognitive function improved more with repeated testing in the exposure groups, but the difference from the control group remained clear. These results suggest that exposure to methylmercury impairs higher brain functions, such as cognitive ability, judgment, and concentration. However, the results also indicate the possibility of plasticity, meaning that functional recovery through rehabilitation may be possible for patients with milder methylmercury poisoning.

Methylmercury causes a “thinning-out” loss of neurons in the cerebral and cerebellar cortices. This suggests that with milder exposure, remaining brain neurons are more functional, and plasticity is stronger. Our results are consistent with the mechanism of methylmercury-induced neurotoxicity.

The limitation of this study is the small sample size. However, it is difficult to conduct similar tests in present-day Minamata because there are few patients of the same age with similar symptoms as those at the time. At that time, our research capabilities were also limited, making it difficult to expand these studies. Nevertheless, we believe it is worthwhile to conduct similar tests on people around the world who have been exposed to methylmercury.

RTs to complex tasks are useful for detecting health effects and dynamic changes in cases involving milder methylmercury exposure or central nervous system damage. In addition to measuring RT, computers can be used to evaluate attention, judgment, memory, and other abilities [9]. Such tests may be useful for identifying early or mild abnormalities in the function of the central nervous system caused by toxic substances.

## Figures and Tables

**Table 1:** Basic information on subjects.

Group	N	Age	Sex	Daily Use of PC	Occupation	Parent’s Occupation	Education (Junior High School/ High School/College)
			(M/F)	(Yes/No)	(Fishery-Related/ Others/Unknown)	(Fishery-Related/ Others/Unknown)	
Group C	12	48.6 ± 7.0	3/9	5/7	0/12/0	0/12/0	2/9/1
Group E-HA	12	48.8 ± 6.0	7/5	4/8	1/10/1	4/7/1	5/7/0
Group E+HA	12	48.8 ± 8.5	4/8	4/8	1/5/6	1/5/6	3/8/1

**Table 2:** Neurological findings (*n* = 12 in each group).

Group	Grade	Visual Constriction	Auditory Disturbance	Dysarthria	Uppe Extremity Ataxia	Lower Extremity Ataxia	Truncal Ataxia
Group E-HA	Severe	0	0	0	0	0	4 (33%)
	Moderate–Mild	0	0	1 (8%)	0	1 (8%)	3 (25%)
	None	12 (100%)	12 (100%)	11 (92%)	12 (100%)	11 (92%)	5 (42%)
Group E+HA	Severe	0	0	0	2 (17%)	0	9 (75%)
	Moderate–Mild	0	0	1 (8%)	10 (83%)	3 (25%)	2 (17%)
	None	12 (100%)	12 (100%)	11 (92%)	0	9 (75%)	1 (8%)

**Table 3:** The number (%) of people whose two-point discrimination threshold was 5 mm or more.

Group	Tongue or Lower Lip	Right Index Finger	Left Index Finger
Group E-HA (*n* = 11)	1 (9%)	3 (27%)	3 (27%)
Group E+HA (*n* = 12)	6 (50%)	9 (75%)	11 (92%)

*p* = 0.0937 in tongue or lower lip, *p* = 0.0613 in right index finger, *p* = 0.0063 in left index finger.

**Table 4:** Frequency of false responses.

Group C 1st test	0.83 ± 1.03
Group C 2nd test	0.33 ± 0.49
Group E-HA 1st test	1.08 ± 1.08
Group E-HA 2nd test	0.83 ± 1.03
Group E+HA 1st test	1.33 ± 1.44
Group E+HA 2nd test	0.67 ± 0.89

There was no statistical difference between groups.

## Data Availability

The data are unavailable due to privacy and ethical restrictions.

## Supplementary Materials

The following supporting information can be downloaded at: https://www.mdpi.com/article/10.3390/toxics13080657/s1, Table S1. Reaction time (when target “Z” was present); Table S2. Reaction time (when target “Z” was absent); Table S3. *p*-value for comparison of number of stimuli; Table S4. *p*-value of comparison between tests with and without target "Z"; Table S5. *p*-value of comparison between first and second tests; Table S6. *p*-value of comparison among three groups.

## Abbreviations

The following abbreviations are used in this manuscript:

RT	reaction time
ms	millisecond
