# Supplementary material for: Visual Search Test for Residents Chronically Exposed to Methylmercury in the Minamata Area
Source: Toxics. 2025 Jul 31;13(8):657. doi: 10.3390/toxics13080657 (PMC12390029; doi:10.3390/toxics13080657)
Supplement: Supplementary file 1 [file toxics-13-00657-s001.zip › toxics-3743501-supplementary.pdf]

Supplementary Tables

Table S1. Reaction time (when target "Z" was present) unit = ms

	Average ± SD		
	2 Letters	6 Letters	10 Letters
Group C 1st test	715 ± 81	803 ± 118	967 ± 259
Group C 2nd test	673 ± 93	758 ± 119	916 ± 309
Group E-HA 1st test	1122 ± 179 **	1429 ± 294 *	1751 ± 348 **
Group E-HA 2nd test	928 ± 160	1191 ± 222	1423 ± 299
Group E+HA 1st test	1271 ± 505 **	1768 ± 857	2000 ± 1137
Group E+HA 2nd test	1080 ± 389	1582 ± 871	1881 ± 916

*p<0.05, **p<0.01 (Table S5)

In all trials, Group C vs. E-HA and Group C vs. E+HA were p<0.01, and Group E-HA vs. E+HA was n.s. (Table S6)

Table S2. Reaction time (when target "Z" was absent) unit = ms

	Average ± SD		
	2 Letters	6 Letters	10 Letters
Group C 1st test	810 ± 121	1098 ± 248 **	1400 ± 348
Group C 2nd test	773 ± 181	916 ± 190	1307 ± 485
Group E-HA 1st test	1160 ± 206	1672 ± 307 *	2516 ± 531 *
Group E-HA 2nd test	1061 ± 256	1438 ± 315	1972 ± 514
Group E+HA 1st test	1454 ± 554 **	2011 ± 915 *	2816 ± 1257 **
Group E+HA 2nd test	1239 ± 452	1762 ± 771	2282 ± 1024

*p<0.05, **p<0.01 (Table S5)

In all trials, Group C vs. E-HA and Group C vs. E+HA were p<0.01, and Group E-HA vs. E+HA was n.s. (Table S6)

Table S3. P-value for comparison of number of stimuli

When "Z" was present	Group C	Group E-HA	Group E+HA
1st test (2) vs. 1st test (6)	0.001**	0.003**	0.009**
1st test (2) vs. 1st test (10)	0.004**	0.000**	0.018**
1st test (6) vs. 1st test (10)	0.022*	0.017*	0.106
2nd test (2) vs. 2nd test (6)	0.016*	0.000**	0.009**
2nd test (2) vs. 2nd test (10)	0.019*	0.000**	0.002**
2nd test (6) vs. 2nd test (10)	0.044*	0.004**	0.120
**When "Z" was absent**	**Group C**	**Group E-HA**	**Group E+HA**
1st test (2) vs. 1st test (6)	0.000**	0.000**	0.010*
1st test (2) vs. 1st test (10)	0.000**	0.000**	0.000**
1st test (6) vs. 1st test (10)	0.000**	0.000**	0.000**
2nd test (2) vs. 2nd test (6)	0.042*	0.000**	0.002**
2nd test (2) vs. 2nd test (10)	0.003**	0.000**	0.001**
2nd test (6) vs. 2nd test (10)	0.002**	0.000**	0.002**

*p<0.05, **p<0.01

Table S4. P-value of comparison between tests with and without target "Z"

	2 Letters	6 Letters	10 Letters
Group C 1st test	0.001**	0.001**	0.000**
Group C 2nd test	0.017*	0.006**	0.002**
Group E-HA 1st test	0.496	0.007**	0.000**
Group E-HA 2nd test	0.021*	0.002**	0.001**
Group E+HA 1st test	0.002**	0.001**	0.001**
Group E+HA 2nd test	0.040*	0.076	0.003**

*p<0.05, **p<0.01

Table S5. P-value of comparison between first and second tests

When "Z" was present	2 Letters	6 Letters	10 Letters
Group C	0.134	0.089	0.202
Group E-HA	0.005**	0.024*	0.000**
Group E+HA	0.009**	0.204	0.407
**When "Z" was absent**	**2 Letters**	**6 Letters**	**10 Letters**
Group C	0.453	0.000**	0.238
Group E-HA	0.071	0.020*	0.011*
Group E+HA	0.005**	0.027*	0.005**

*p<0.05, **p<0.01

Table S6. P-value of comparison among three groups

First test, "Z"(+)	2 Letters	6 Letters	10 Letters
Group C vs. E-HA	0.000**	0.000**	0.000**
Group C vs. E+HA	0.001**	0.001**	0.006**
Group E-HA vs. E+HA	0.344	0.209	0.476
**Second test, "Z"(+)**	**2 Letters**	**6 Letters**	**10 Letters**
Group C vs. E-HA	0.000**	0.000**	0.000**
Group C vs. E+HA	0.002**	0.004**	0.002**
Group E-HA vs. E+HA	0.224	0.146	0.114
**First test, "Z"(-)**	**2 Letters**	**6 Letters**	**10 Letters**
Group C vs. E-HA	0.000**	0.000**	0.000**
Group C vs. E+HA	0.001**	0.003**	0.001**
Group E-HA vs. E+HA	0.099	0.237	0.454
**Second test, "Z"(-)**	**2 Letters**	**6 Letters**	**10 Letters**
Group C vs. E-HA	0.004**	0.000**	0.004**
Group C vs. E+HA	0.003**	0.001**	0.007**
Group E-HA vs. E+HA	0.247	0.191	0.359

*p<0.05, **p<0.01
